# A Literature-Based Update on *Benincasa hispida* (Thunb.) Cogn.: Traditional Uses, Nutraceutical, and Phytopharmacological Profiles

**DOI:** 10.1155/2021/6349041

**Published:** 2021-12-10

**Authors:** Muhammad Torequl Islam, Cristina Quispe, Dina M. El-Kersh, Manik Chandra Shill, Kanchan Bhardwaj, Prerna Bhardwaj, Javad Sharifi-Rad, Miquel Martorell, Rajib Hossain, Ahmed Al-Harrasi, Ahmed Al-Rawahi, Monica Butnariu, Lia Sanda Rotariu, Hafiz Ansar Rasul Suleria, Yasaman Taheri, Anca Oana Docea, Daniela Calina, William C. Cho

**Affiliations:** ^1^Department of Pharmacy, Life Science Faculty, Bangabandhu Sheikh Mujibur Rahman Science and Technology University, Gopalganj (Dhaka) 8100, Bangladesh; ^2^Facultad de Ciencias de la Salud, Universidad Arturo Prat, Avda. Arturo Prat 2120, Iquique 1110939, Chile; ^3^Pharmacognosy Department, Faculty of Pharmacy, The British University in Egypt (BUE), El Sherouk, Cairo Governorate, Egypt; ^4^Department of Pharmaceutical Sciences, North South University, Bashundhara, Dhaka 1229, Bangladesh; ^5^Department of Botany, Shoolini University of Biotechnology and Management Sciences, Solan-173229, H. P., India; ^6^Facultad de Medicina, Universidad del Azuay, Cuenca, Ecuador; ^7^Department of Nutrition and Dietetics, Faculty of Pharmacy, and Centre for Healthy Living, University of Concepción, 4070386 Concepción, Chile; ^8^Natural and Medical Sciences Research Centre, University of Nizwa, Birkat Almouz, 616, Oman; ^9^Banat's University of Agricultural Sciences and Veterinary Medicine “King Michael I of Romania” from Timisoara, 300645, Calea Aradului 119, Timis, Romania; ^10^Department of Agriculture and Food Systems, The University of Melbourne, Australia; ^11^Phytochemistry Research Center, Shahid Beheshti University of Medical Sciences, Tehran, Iran; ^12^Department of Toxicology, University of Medicine and Pharmacy of Craiova, 200349 Craiova, Romania; ^13^Department of Clinical Pharmacy, University of Medicine and Pharmacy of Craiova, 200349 42 Craiova, Romania; ^14^Department of Clinical Oncology, Queen Elizabeth Hospital, Kowloon, Hong Kong

## Abstract

*Benincasa hispida* (Thunb.) Cogn. (Cucurbitaceae) is an annual climbing plant, native to Asia with multiple therapeutic uses in traditional medicine. This updated review is aimed at discussing the ethnopharmacological, phytochemical, pharmacological properties, and molecular mechanisms highlighted in preclinical experimental studies and toxicological safety to evaluate the therapeutic potential of this genus. The literature from PubMed, Google Scholar, Elsevier, Springer, Science Direct, and database was analyzed using the basic keyword “*Benincasa hispida*.” Other searching strategies, including online resources, books, and journals, were used. The taxonomy of the plant has been made by consulting “The Plant List”. The results showed that *B. hispida* has been used in traditional medicine to treat neurological diseases, kidney disease, fever, and cough accompanied by thick mucus and to fight intestinal worms. The main bioactive compounds contained in *Benincasa hispida* have cytotoxic, anti-inflammatory, and anticancer properties. Further safety and efficacy investigations are needed to confirm these beneficial therapeutic effects and also future human clinical studies.

## 1. Introduction

Food and food products are being used as medicines over centuries worldwide. Many species from the family Cucurbitaceae have been used as medicaments in various diseases in Ayurveda and ancient Chinese medicine. This family is also known as the gourd family. It provides approximately 5 to 6% of the total vegetables in the world. To date, 825 species from under 118 genera have been reported growing in temperate regions of the world [1]. It should be mentioned that the Cucurbit species can grow in diverse climatic conditions, including arid deserts, tropical, subtropical, and temperate regions. These various types of species are included in food systems and Indian traditional medicines. Generally, the gourd family vegetables provide vitamins, essential minerals, antioxidants, and soluble fibres [2].

The word “herb” derived from the Latin word “*herba*” and an old French word “*herbe*” refers to any part of the plant like fruit, seed, stem, bark, flower, leaf, stigma, or a root, as well as a non-woody plant. Many herbs are currently under-using as a source of foods, flavonoids, medicines, or perfumes as well as in certain spiritual activities. Ancient era literature including Unani manuscripts, Chinese writings, and Egyptian papyrus also depicted the use of herbs in various diseases. The Indian Vaids, Unani Hakims, and European and Mediterranean cultures are using herbs for more than 4000 years as medicines. Native people of Iran, Rome, Egypt, Africa, and America used medicinal herbs in healing habits. The Unani, Ayurveda, and Chinese Medicine are using herbal remedies systematically. These all are the potential sources of medicinal plant-based modern medicines. According to World Health Organization (WHO), about 80% of people in the world depend on herbal medicines to fulfil their basic health care needs, and around 21,000 species of plants have been identified as potential medicinal plants. In developed countries, around 25% of the total drugs come from plant origin, while in fast-developing countries as much as 80% [3].

*Benincasa hispida* (Thunb.) Cogn. (synonym: *Benincasa cerifera* Savi) (Cucurbitaceae) especially in Asian countries is considered as one of the famous crops under the Cucurbitaceae family that grows mainly for its fruits and well renowned for its nutritional and medicinal properties [4, 5]. Scientific reports suggest that *B. hispida* possesses many important nutritious substances, including vitamins, natural sugars, amino acids, organic acids, and mineral elements [4, 6, 7]. This review is aimed at sketching an up-to-date scenario on the indigenous uses, nutraceutical, and phytochemical composition along with the pharmacological activities of *B. hispida* based on database reports.

## 2. Review Methodology

Using the PubMed database and the search engines Google scholar, Elsevier, Springer, Science Direct, research articles, and reviews related to *B. hispida* were analyzed. Abstracts and papers peer reviewed were selected according to the objectives of the research: the molecular pharmacological mechanisms of action proven by preclinical experimental studies [8] and which scientifically justify the traditional uses of *B. hispida.*

Other sources of “grey literature” information such as Web pages, book chapters, and specialized monographs were also analyzed to obtain maximum updated information on the biological properties of this plant. The keywords used were “*Benincasa hispida*” or “traditional uses” or “phytochemistry” of “pharmacological properties” or “biological activities” or “toxicology” or “safety” or “side effects.” The scientific names of the plants were verified according to PlantList, and the chemical formulas were revised by consulting the PubChem database (https://scholar.google.com).

Inclusion criteria: the most relevant articles written in English on taxonomy, ethnopharmacology, phytochemistry, pharmacology, various biological activities, and toxicity of this plant were included and analyzed.

Exclusion criteria: the papers containing homoeopathic preparations, papers written in languages other than English, publications without pharmacological mechanisms of action.

## 3. Botany and Traditional Uses

### 3.1. Botany (Plant Profile)

**Figure 1:**
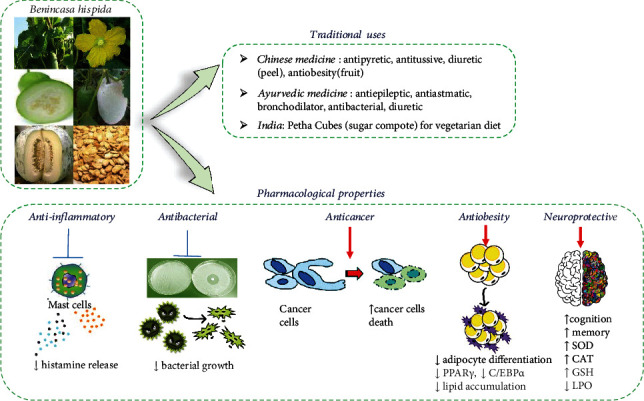
Diagram with different parts (aerial parts, flower, fruit pulp unripe, fruits, fruit pulp mature, and seeds) of *Benincasa hispida* (Thunb.) Cogn, traditional uses, and its most important pharmacological properties. Abbreviations: PPAR*γ*: peroxisome proliferator-activated receptor gamma; C/EBP*α*: CCAAT enhancer-binding protein alpha; CAT: catalase; SOD: superoxidase dismutase; GSH: reduced glutathione; LPO: lipid peroxidations.

*B. hispida* (Figure 1), also known as *kundur fruit*, *chalkumra*, *wax gourd*, *winter gourd*, *ash gourd*, *winter melon*, *white gourd*, *tallow gourd*, *Chinese preserving melon*, *ash pumpkin*, and *(alu) puhul*, a creeper grown for its very big size fruit, is eaten as a green mature vegetable or greens [9–11].

There is a fine hairs fuzzy coating outer side of the young fruit and has solid thick white flesh of sweet in tastes. The mature fruit sheds its hairs and forms a waxy white coating, giving the name of “*wax gourd*.” The gourd wax coating increases the storage facilities of it. It can grow of a length up to 80 cm and also have broad leaves and yellow flowers. The taste is rather bland. *B. hispida* is a native of South and Southeast Asia. However, it is commonly grown all over Asia, including Japan, Burma, Ceylon, Sri Lanka, Java, and Australia [10].

Taxonomy

*Kingdom*: Plantae

*Phylum*: Tracheophyta

*Class*: Magnoliopsida

*Oder*: Cucurbitales

*Family*: Cucurbitaceae

*Genus*: Benincasa

*Species*: *Benincasa hispida* (Thunb.) Cogn.

### 3.2. Traditional Uses and Ethnopharmacology

In India, *B. hispida* is used as a winter season vegetable for a wide variety of diseases. Its medicinal properties have been also recognized in the Ayurvedic system of medicine, spiritual traditions of India and Yoga. In Vietnam, its soup (cooked with pork short ribs) is traditionally used by breastfeeding mothers. In north India and almost all regions in Bangladesh, it is added with pulses like as moong which usually crushed, along with wax gourd, makes a dish locally called *bori*, which after sun drying is used in curry dishes and eaten with rice or chapati [12]. To make wax gourd soup in China, it is used in stir-fries or added into pork or pork/beef bones, which often served in the scooped-out gourd, carved by scraping off the waxy coating. It is also cut into pieces, candied and normally eaten during the time of New Year festivals, or used as filling in Sweetheart cake. For the Moon Festival, the Chinese and Taiwanese also used it in moon cakes as a base filling. It is candied by the people of the Philippines and is used as a pastry filling for bakpia. In some savoury soups and stir-fries, it also acts as an ingredient. In Nepal, India, and Bangladesh, the tendrils, shoots, and leaves of the plant are consumed as green vegetables [6].

*B. hispida* is widely used in Chinese medicine, in the treatment of fever, cough accompanied by thick mucus and urinary disorders, it is used especially in bark with a very good diuretic effect. The fruit is recommended for overweight people who want to follow diets. In Ayurvedic medicine, it is used in the treatment of epilepsy, cough, lung disease, hiccups, asthma, internal bleeding, and urinary retention. In India, a fruit compote called Petha Cubes is made from the pulp of the fruit, which is recommended for vegetarians [13].

The fruit is also used in peptic ulcer, and it is also used in diabetes mellitus, urinary infection, haemorrhages from internal organs, insanity, epilepsy, and other nervous disorders in Ayurveda [14]. The fruit is sweet and traditionally used as a cooling, styptic, antiperiodic, laxative, diuretic, tonic, aphrodisiac, and cardiotonic, and also in jaundice, dyspepsia, urinary calculi, blood disease (e.g., haemorrhages from internal organs), insanity, epilepsy, asthma, diabetes, vitiated conditions of pitta, fever, menstrual disorders, and balancing the body heat [15] (Figure 1).

### 3.3. Phytochemical Profile

#### 3.3.1. Nutritional Composition

The edible portion of *B. hispida* contains moisture (93.80-96.80/100 g), proteins (0.30-0.70/100 g), carbohydrates (1.10-4.00/100 g), fat (0.02-0.20/100 g), fibre (0.50-2.10/100 g), and ash (0.27-0.70/100 g).

Vitamins present in the edible portion (per 100 g) of this plant are vitamin C (1.35-68.00), thiamin (0.02-0.04), riboflavin (0.02-0.31), niacin (0.20-0.46), and vitamin E. Major minerals in the edible portion (per 100 g) include sodium (0.14-6.00), potassium (77.00-131.00), calcium (5.00-23.32), iron (0.20-0.49), and phosphorus (225.39-234.61) [6, 16, 17]. The fruit contains water-soluble polysaccharides [18], such as arabinogalactans [19].

The fruit pulp contains homogalacturonan, *β*-(1 → 4)-D-galactan, acidic arabinan [20], and natural sugars (e.g., glucose and fructose) [21]. The mature fruit also contains organic acids such as malic and citric acid.

#### 3.3.2. Chemical Phytoconstituents

**Figure 2:**
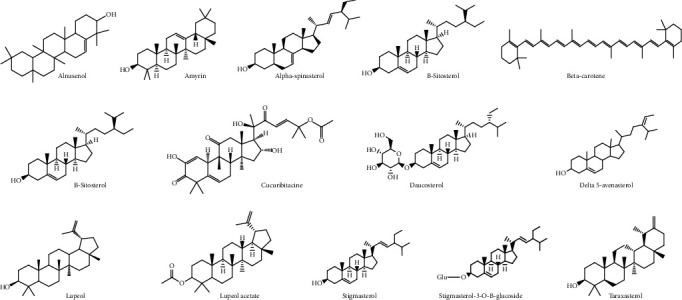
The chemical formulas of most important representative sterols and terpenes from *Benincasa hispida*.

The leaf contains alkaloids, flavonoids, steroids [22], and the fruit amino acids, pectic polysaccharides [20], hemicellulose polysaccharides [18], terpenes and terpenoids, flavonoid C–glycosides, sterols [23], proteins [24], phenols, alkaloids, glycosides, tannins, saponins [25], hydroxybenzoic acids, flavonols, hydrocinnamic acids, and triterpenes [9] (Figure 2).

The seeds contain proteins [24], carbohydrates, phenolic compounds, amino acids, flavonoids, sterols [26], glycosides, alkaloids, fixed oils and fats, phenolic compounds, steroids [27], and unsaturated fatty acids [28]. The peel contains alkaloids, saponins, steroids, carbohydrates, flavonoids [29], tannins, carotenoids, oxalates, and phytate [17].

The root contains proteins [24]. The fruit contains many volatile compounds, including (*E,E*)-2,4-nonadienal, (*E*)-2-hexenal, n-hexanal, n-hexyl formate, (*E,E*)-2,4-heptadienal, (*Z*)-3-hexenal, (*E*)-2-heptenal, 1-octen-3-ol [30], 2,5-dimethylpyrazine, 2-methyl pyrazine, 2-ethyl-5-methyl pyrazine, and 2,6-dimethylpyrazine, 2,3,5-trimethylpyrazine [30].

**Figure 3:**
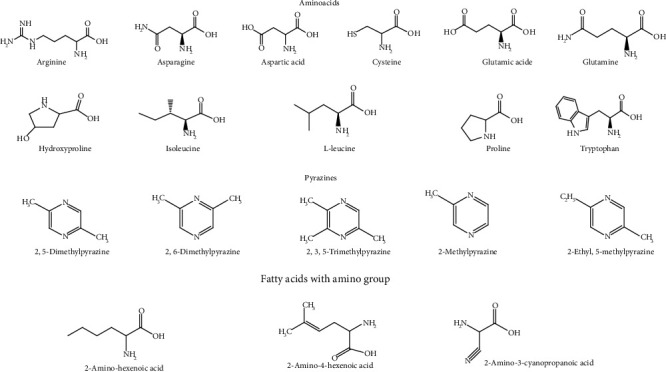
The chemical formulas of most important representative carbohydrates from *Benincasa hispida*.

**Figure 4:**
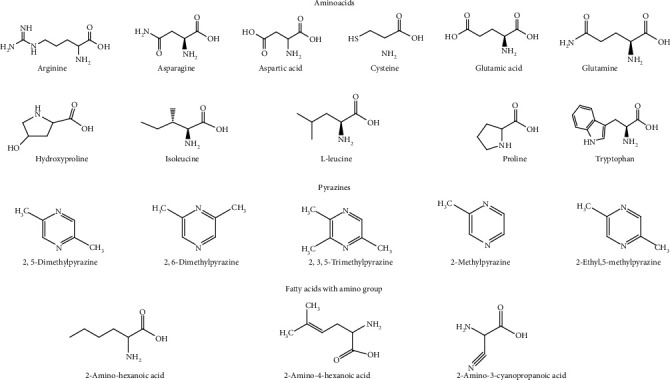
The chemical formulas of most important representative aminoacids, pyrazines, and fatty acids with amino group from *Benincasa hispida*.

**Figure 5:**
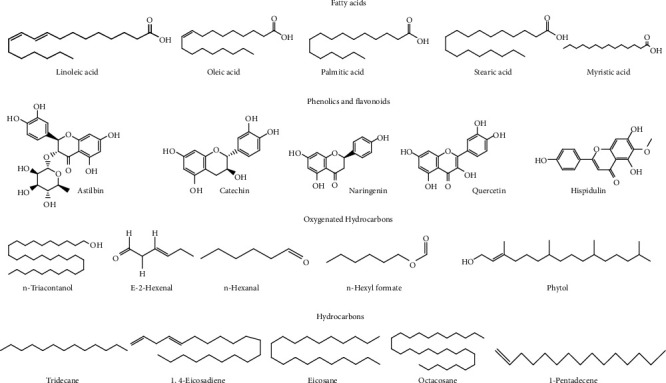
The chemical formulas of most important representative fatty acids, phenolic and flavomoids, oxygenated hydrocarbons, and hydrocarbons from *Benincasa hispida*.

*B. hispida* is rich in phenolic compounds. Several other bioactive compounds present in it are isomultiflorenyl acetate, isovitexin, 1-sinapoylglucose, multiflorenol, 5-gluten-3-*β*-ylacetate, alnusenol, and benzylalcolcohol-*O*-*α*-l-arabinopyranosyl-(1-6)-*β*-d-glucopyranoside [31]. The most representative phytochemicals present in *B. hispida* has been shown in Figures 2–5 and Table 1.

## 4. Pharmacological Activities

### 4.1. Antioxidant Effects

Oxidative stress is a term used for free radical diseases [51, 52]. It is defined as the imbalance between free radicals and antioxidants, given that oxidants (free radicals) are more and have a destructive potential on the human body [53, 54].

The methanolic seed extract showed a concentration-dependent (25-200 *μ*g/mL) 2,2-diphenyl-1-picrylhydrazyl (DPPH) and hydrogen peroxide radical scavenging effects [55]. Another study revealed that the ethanolic seed extract shows better DPPH and 2,2′-azino-bis(3-ethylbenzothiazoline-6-sulfonic acid (ABTS) radical scavenging along with total phenolic content (TPC) than its ethyl acetate and n-hexane extracts [43]. The seed oil (0.1 mg/mL) also showed significant DPPH and ABTS radical scavenging capacity [56]. This study also determined the TPC in seed oil. The aqueous extract of this plant reduced reactive oxygen species (ROS) in human umbilical vein endothelial cells (HUVECs) [57].

Polysaccharides of fruit extract showed DPPH free radicals scavenging activity with an EC_50_ value of 0.98 mg/mL [50]. The seed oil also showed DPPH and ABTS radical scavenging capacity. However, the antioxidant activity was lower than the catechin and BHT at the same concentration (0.1 mg/mL) [44]. Petroleum ether and methanol fruit extracts increased in catalase (CAT) levels in gastric ulcer rats [58]. Hispidalin isolated from this herb also showed DPPH radical scavenging and inhibition of lipid peroxidation capacity [59]. The aqueous fruit extract significantly increased the antioxidant status as well as levels of vitamin C concentration in gastric juice or rats [60].

Antioxidant effects of various parts of *B. hispida* on various test models have been also observed by several authors [17, 28, 29, 56, 61].  Table 2 shows the antioxidant effects of various parts of *B. hispida*.

### 4.2. Anti-Inflammatory Effect

The methanolic seed extract (100-300 mg/kg, p.o.) showed dose-dependent anti-inflammatory effects on carrageenan-induced paw oedema rat (*n* = 6) model [55]. The fruit peel methanolic extract showed an anti-inflammatory effect on egg albumin-induced inflammation in rats [62]. The petroleum ether and methanolic fruit extract of *B. hispida* (300 mg/kg, p.o.) showed a dose-dependent anti-inflammatory effect on cotton pellet-induced granuloma models in rats, carrageenan-induced paw oedema, and histamine-induced paw oedema [58].

### 4.3. Antimicrobial, Antihelmintic, and Larvicidal Effects

Due to the excessive use of antibiotics that can lead to the development of antibiotic resistance of various strains of bacteria [63–65], attempts have been made to use natural antibiotic alternatives [66, 67]. Most of these options include plants with antiviral and antibacterial properties that can be effective against gram-negative and gram-negative germs, which are often difficult to eradicate [68, 69]. The methanolic whole plant extract (500 *μ*g/disc) was found to act against *Pseudomonas aeruginosa and Vibrio parahaemolyticus* [70]. In the latter case, the zone of inhibition was 6 mm only. Hispidalin, an isolated compound from this herb, was found to act against several bacteria (e.g., *Escherichia coli*, *Pseudomonas aeruginosa*, *Staphylococcus aureus*, and *Salmonella enterica*) and fungi (e.g., *Aspergillus flavus*, *Penicillium chrysogenum*, *Fusarium Solani*, and *Colletotrichum gloeosporioides*) *[*59*]*. In this case, the minimum inhibitory concentrations (MIC) were 30-120 and 100-200 *μ*g/mL for bacterial and fungal strains, respectively. Moreover, the aqueous, methanol, and petroleum extracts of seeds showed significant therapeutic efficacy with methanol extract being the best comparable to the antibiotic ciprofloxacin. In other study, the aqueous peel extract showed strong antibacterial activity against *S. aureus* (MIC = 14.5 *μ*g/mL), *Micrococcus luteus* (MIC = 8.6 *μ*g/mL), *E. coli* (MIC = 6.1 *μ*g/mL), and *Klebsiella pneumoniae* (MIC = 13.4 *μ*g/mL) [11]. The herb shows prebiotic activity [71].  Table 3 shows the antimicrobial effects of various parts of *B. hispida*.

Ethanolic seed extract (20, 40 and 60 mg) showed a dose-dependent anthelmintic in anthelmintic activity on *Pheretima posthuma* [72]. The phloem lectin-like protein from the exudate of the herb exerted an inhibitory effect on the *Samia ricini* larvae [46] (Table 3).

### 4.4. Cytotoxic and Anticancer Effects

Cancer is a term used to define malignancies in which abnormal cells multiply in an uncontrolled and continuous manner and can invade the surrounding healthy tissues [73, 74]. Abnormal cells come from any tissue in the human body and can occur anywhere in the body [75–77]. Natural anticancer alternatives can have a direct effect on malignant cells, as well as by stimulating the body's immune capacity in the fight against the aggression of carcinogenic factors, internal or external [78, 79]. The favourable effects of some medicinal plants are due to the main biochemical components: flavonoids—which inhibit the activity of carcinogens and prevent the metastasis of malignant cells; carotenoids—which protect the body against colon cancer; terpenes in essential oils—block the action of carcinogens, having a strong antioxidant action; *β*-carotene, a powerful antioxidant with anticancer protection and a recognized inhibitor of malignant cells; antioxidant vitamins C, E, and A, destroy free radicals, prevent cancer, and block the metastasis process [80–83].

**Figure 6:**
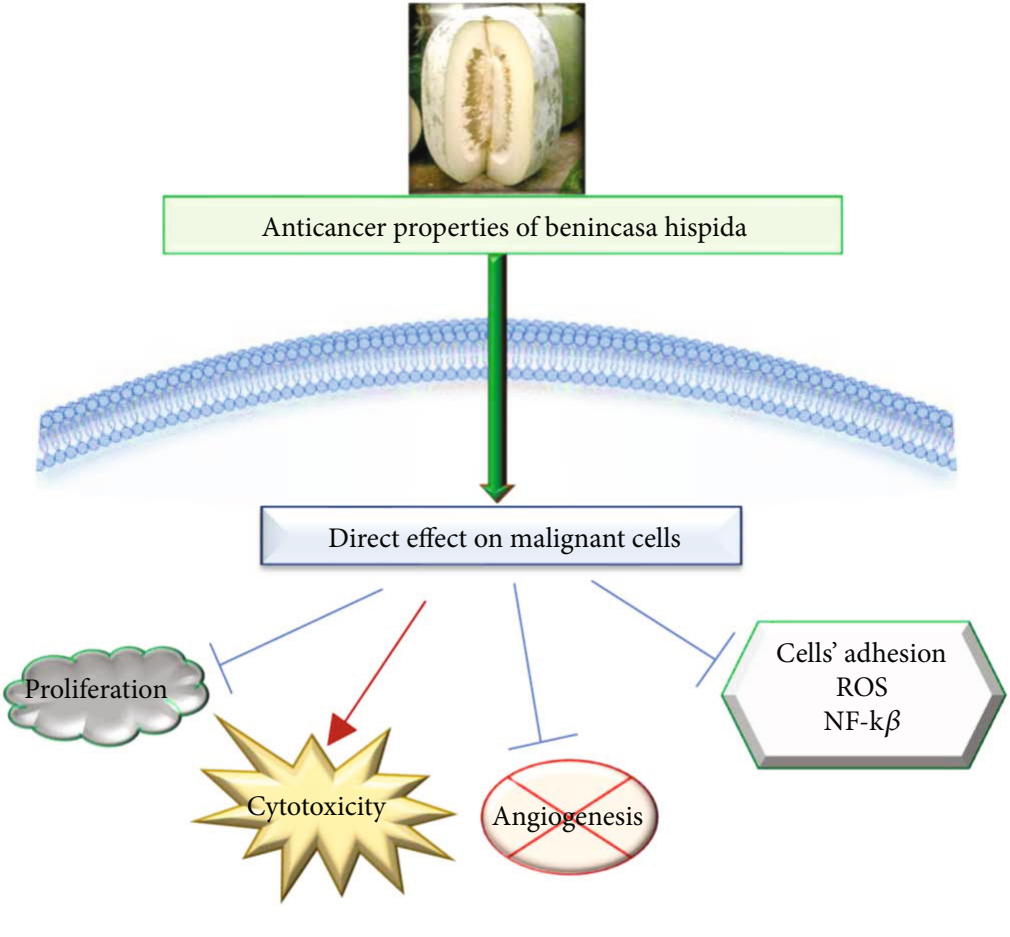
A schematic diagram with anticancer mechanisms of natural compounds from *Benincasa hispida*. Legend: blue arrow: inhibition, reduction; red arrow: increase, stimulation, ROS reactive oxygen species, NF*-κ*B (nuclear factor kappa-light-chain-enhancer of activated B cells).

The fruit, seed, and root proteins (10-1000 *μ*g/mL) exerted a concentration-dependent cytotoxic effect on *Artemia salina.* The median lethal concentration (LC_50_) values of fruit, seed, and root extract were 44, 41, and 50 *μ*g/mL, respectively [24]. In this study, the root proteins inhibited the proliferation of HeLa and K-562 cells by 28.50 and 36.60%, respectively. Another study reveals that the whole plant methanolic extract *(5-50 μ*g/mL) exerted a cytotoxic effect on *A. salina* (LC_50_: 45.187 *μ*g/mL) [70]. Moreover, the aqueous seed extract (20-800 *μ*g/mL) did not exert cytotoxic effects on HUVECs and normal fibroblast (NIH/3T3) cells. On male C57BL/6 mice, the extract showed a potent inhibitory effect on basic fibroblast growth factor- (bFGF-) induced angiogenesis [84]. The aqueous extract (1–20 *μ*g/mL) also reduced cell adhesion molecules activation by inhibiting monocyte adhesion, ROS, and nuclear factor kappa-light-chain-enhancer of activated B cells (NF-*κ*B) on high glucose (25 mM) induced HUVECs cells [57] (Figure 6).

Table 4 shows the cytotoxic and anti-cancer effects of various parts of *B. hispida*, and Figure 1 summarizes the most important anticancer mechanism.

### 4.5. Gastrointestinal Protective Effects

#### 4.5.1. Gastroprotective Effect

Fresh juice (1-4 mL/animal, p.o.), ethanol (12, 24 and 48 mg/kg, p.o.), and pet ether extract (0.75, 1.5 and 3 mg/kg, p.o.) in swimming stress, aspirin plus restraint, serotonin-induced ulcers, and indomethacin plus histamine displayed a dose-dependent antiulcerogenic effect in rats and mice [13].

The petroleum ether and methanol fruit extracts (300 mg/kg, p.o.) significantly (*P* < 0.05) reduced ulcer index, vascular permeability, and malondialdehyde (MDA) content, while an increase in CAT levels in comparison to the control group in pylorus ligated (PL) gastric ulcers, ethanol-induced gastric mucosal damage, and cold restraint stress- (CRS-) induced gastric ulcer rat models [58]. The fruit extract (1 mL/kg, p.o.) also decreased ulcer index as well as MDA, superoxide dismutase (SOD), and vitamin C levels in indomethacin-induced gastric ulcer in rats [23].

The hydromethanol, ethyl acetate, and aqueous ripe fruit extracts (20 mg/kg, p.o./alternative days) were treated for 14 days in ranitidine (5 mg/kg, p.o.) induced hypochlorhydria in rats. The aqueous extract showed better effects on the test animals. It increased the antioxidant status as well as levels of pepsin, vitamin C, and gastric juice chloride concentration than the other extracts [60]. On the other hand, the extract of fruits with the whole plant of *Fumaria vaillantii* Loisel (1 : 1) (20 mg/kg, p.o.) was administrated in ranitidine (5 mg/kg) induced hypochlorhydria in rats as pre-and cotreatment manners. The extract significantly (*P* < 0.05) enhanced the concentration of pepsin, iron levels in serum, chloride level in gastric juice, and liver along with blood haemoglobin level in experimental animals [85].

A prospective pilot study on dyspeptic patients (*n* = 20) (baseline between 30 days and 45 days) aged between 18 and 45 years with only single dose of 200 mL fruit juice every morning in empty stomach for thirty days suggests that a significant improvement of pain, nausea, belching, retrosternal burning, and bowel habits among the patients [86].  Table 5 shows the gastrointestinal -protective effects of various parts of *B. hispida*.

#### 4.5.2. Antidiarrheal Effect

Diarrhoea is a condition characterized by frequent watery stools, and usually, diarrhoea persists for a few days and is treated with diet [87]. But there are also more serious situations, in which diarrhoea requires drug/complementary treatment and is more difficult to cure [88, 89].

The *B. hispida* fruit methanolic extract displayed potential antidiarrheal activity on the castor oil-induced diarrheal rat model. It was also seen to inhibit induced PGE2, enter pooling, and reduce in the motility of gastro-intestine in charcoal meal rats [90]. The same extract also possessed a significant inhibitory activity against castor oil-induced diarrhoea and induced PGE2, enter pooling and gastrointestinal motility at 200, 400, and 600 mg/kg (p.o.) in castor oil, charcoal meal, and antienter pooling models in rats [90] (Table 5).

### 4.6. Effects on Metabolic Diseases

#### 4.6.1. Antidiabetic Effects

The methanolic stem extract (50,100, 200 mg/kg, p.o.) dose-dependently lowered the blood glucose level in alloxan-induced diabetic rats [37]. The chloroform fruit extract (250 and 500 mg/kg, p.o.) dose-dependently ameliorated the derangements in lipid metabolism in alloxan-induced diabetic albino rats after 14 days of treatment [91]. The study reveals that the methanol, ethanol, and aqueous peel extracts showed significant *α*-amylase inhibition activity [17]. The ethanol and ethyl ethanoate leaf extracts lowered the blood glucose level of the diabetic mice in a dose-dependent manner [22]. Antidiabetic effects of various parts of *B. hispida* have been shown in Table 6.

#### 4.6.2. Antiobesity and Lipid-Lowering Effect

Lipids are fatty organic substances that are the largest source of energy for the body. The vast majority of fats are stored in solid form in various organs or skin, and a small part circulates in the blood in liquid form [92, 93]. Imbalances in lipid metabolism lead to pathophysiological changes and the appearance of chronic diseases such as cardiovascular disease, fatty liver, endocrine disorders, and diabetes [94, 95]. Methanolic fruit extract (0.2-1 g/kg, i.p.) reduced food intake, suggesting anorectic activity in mice [96]. Hexane fraction from the aqueous fruit extract inhibited adipocyte differentiation by blocking leptin gene expression, peroxisome proliferator-activated receptor gamma (PPAR*γ*), and CCAAT enhancer-binding protein alpha (C/EBP*α*), resulting in the reduction of lipid accumulation, increased releasing of glycerol and intracellular triglycerides in 3T3-L1 cells [97].

### 4.7. Neuroprotective Properties

#### 4.7.1. Anticonvulsant Effects

The fruit methanol extract (0.2-1 g/kg, p.o.) showed a dose-dependent anticonvulsant activity in pentylenetetrazole, strychnine and picrotoxin, and maximal electro seizures model [98]. On the other hand, the fruit peel methanolic extract exerted a dose-dependent (0.25-1.5 g/kg) anticonvulsant effect on pentylenetetrazol-induced convulsion in mouse models [62]. Ethanolic seed extract (250 and 500 mg/kg, p.o.) showed a dose-dependent anticonvulsant effect in anticonvulsant activity in Swiss albino mice [72].

#### 4.7.2. Effects on Alzheimer's Disease

Neurodegenerative diseases such as Alzheimer's disease are characterized by the presence of the central nervous system, protein aggregates, inflammation, and oxidative stress [99, 100]. Several factors are involved in triggering neurodegenerative diseases, including the lifestyle that leads to the gradual deterioration of the health of the nervous system, with serious consequences on the quality of life of the patient with such a disease [101]. Although there are still no treatment solutions to restore nerve function in neurodegenerative diseases, more and more studies insist on several natural formulas that have been shown to have the effect of reducing symptoms and improving the quality of life of patients with neurodegenerative diseases [102, 103].

The fruit extract at a dose of 400 mg/kg (p.o.) showed a protective effect on colchicine-induced Alzheimer's disease rats, possibly through the presence of both vitamin E and *β*-carotene protecting rat neurons against oxidative stress. On the other hand, the aqueous fruit pulp extract (100-450 mg/kg, p.o.) dose-dependently increased SOD, CAT, and GSH, while reduced in LPO levels in the colchicine-induced Alzheimer's rat model [16].

#### 4.7.3. Effects on Memory and Cognitive Behaviour

Cognitive disorders are characterized by changes in brain structure and function that affect learning, orientation, judgment, memory, and intellectual abilities [104–106]. The methanolic fruit extract (200, 400, or 600 mg/kg, p.o.) showed a significant dose-dependent anticompulsive effect in marble-burying and motor coordination test models in mice [38]. The petroleum ether, methanolic, and aqueous fruit extracts (100, 200, and 400 mg/kg, p.o.) showed a dose-dependent nootropic activity in the cognitive behaviour mouse model [107]. Kumar and Nirmala [108] also studied the possible nootropic effects of the fruit on experimental animals.

#### 4.7.4. Antidepressant and Anxiolytic Effects

Anxiety is defined as a diffuse fear, without a well-defined cause regarding various events of daily life [109]. Methanolic fruit extract (50, 100, and 200 mg/kg, p.o.) showed a dose-dependent antidepressant-like effect in TST and FST models possibly through GABAergic involvement in mice in Swiss mice [110].

Petroleum ether, methanolic, and aqueous fruit extracts (100, 200, and 400 mg/kg, p.o.) confirmed a dose-dependent anxiolytic activity in mice [107]. Effects of various parts of *B. hispida* on the nervous system have been shown in Table 6.

### 4.8. Analgesic and Antipyretic Effects

The methanolic fruit extract (200, 400, and 600 mg/kg, p.o.) showed a dose-dependent analgesic effect in acetic acid-induced writhing and hot plate model in mice [111]. The ethanolic seed extract (250 and 500 mg/kg, p.o.) exerted a dose-dependent analgesic effect in rats [26]. The fruit peel methanolic extract also dose-dependently (0.25-1.5 g/kg) inhibited acetic acid-induced writhing, formalin-induced pain licking, and hot plate-induced pain in mice [62].

In another study, the methanolic seed extract (100-300 mg/kg, p.o.) also showed a dose-dependent analgesic effect on the rats (*n* = 6) model [55]. The methanolic leaf extract (50-400 mg/kg, p.o.) exerted a dose-dependent analgesic effect in an acetic acid-induced writhing mouse model [112]. Petroleum ether, methanolic, and aqueous fruit extracts (100, 200 and 400 mg/kg, p.o.) showed a dose-dependent analgesic effect in the mouse model [107]. The fruit juice (1 mL, p.o.) prevents morphine addiction development along with the suppression of opioid withdrawal symptoms [113]. In experimental animals such as rats, mice, and guinea pigs, the methanolic fruit extract (200-3000 mg/kg, p.o.) significantly potentiated the barbiturate stimulated hypnosis [114].

Qadrie et al. [26] reported that the ethanolic seed extract (250 and 500 mg/kg, p.o.) displayed a dose-dependent antipyretic effect in rats.

### 4.9. Other Potential Biological Activities

#### 4.9.1. Bronchodilatator Effect

Fruit methanol extract inhibited histamine release. In this study, two triterpenes, the triterpenes and sterols, multiflorenol and alnusenol exerted better inhibitory effects [33]. The methanolic extract (50, 200, and 400 mg/kg, p.o.) of *B. hispida* exhibited significant protection in guinea pigs against the histamine and acetylcholine-induced bronchospasm [115]. The methanolic fruit extract (200-3000 mg/kg, p.o.) showed significant antihistaminic activity on experimental animals (e.g., rats, mice, and guinea pigs) [114].

#### 4.9.2. Antihypertensive Effect

The ACE inhibitory effect of the plant may show the pharmacological basis in the treatment of high blood pressure for its long time uses in traditional Chinese medicine. The fruit juice (0.4 – 1.6 mL/kg, i.v.) dose-dependently lowered blood pressure, concentration-dependently showed relaxation of isolated rat aortic rings and produced nitric oxide (NO) from the cultured porcine aortic endothelial cells [116]. Polysaccharides of fruit extract showed an antiglycation effect [50].

#### 4.9.3. Nephroprotective Effects

Methanolic fruit extract (500 mg/kg/day, p.o.) for five days reduced the MDA content, while the increase in SOD, CAT, and GSH levels in renal ischemia/reperfusion injury in female Wistar albino rats [117]. The seed ethanolic extract (250 and 500 mg/kg, p.o.) for 35 days significantly lowered the increased urinary oxalate, presenting a regulatory action on endogenous oxalate synthesis; decreased in the urinary excretion and kidney retention levels of protein, oxalate, and calcium; and reduced the increased serum levels of sodium, calcium, phosphorus, and creatinine levels in ethylene glycol induced chronic hyperoxaluria in Wistar albino rat [118].

Effects of various parts of *B. hispida* on the kidney have been shown in Table 6.

#### 4.9.4. Antiageing of Skin

A cream prepared from the dried fruit pulp extract (petroleum ether, chloroform, ethyl acetate, and methanol) showed a significant antiageing effect on the stratum corneum of human skin and dansyl chloride fluorescence models [12].

## 5. Toxicological Profile: Safety and Adverse Effects

The fresh juice (5% *v*/*v*) treatment for 3 months did not change the total white blood cells (WBC), red blood cells (RBC), haemoglobin (HB), mean corpuscular haemoglobin (MCH), hematocrit (HCT), mean corpuscular volume (MCV), sugar, and urea levels in rats and mice. The treatment also caused no behavioural changes in experimental animals [13]. The methanolic extract of fruit was nontoxic and did not cause the death of mice, rats, and guinea pigs in doses up to 3.0 g/kg [114]. Other studies, performed in female and male rats, concluded that the standardized hydroalcoholic (70% ethanol) extract of the fruit pulp of *B. hispida* administered orally was relatively safe when to female and male rats [119]. Up to oral dose (1000 mg/kg body weight/day) level, no-observe-adverse-effect-level (NOAEL) was obtained for the extract in the 90-days toxicity study. The ethanolic seed extract up to 5000 mg/kg (p.o.) did not exert toxicity in rats [26]. Di-2-Ethylhexyl phthalate (18.3-75.5 mg/kg), isolated from the fruit of this herb, is a popularly used plasticizer and is harmful to human health [36].

## 6. Conclusions and Future Perspectives

*Benincasa hispida* (Cucurbitaceae) is an annual plant, originating in Indonesia. The Chinese have been cultivating it for over 2000 years; its medicinal uses first appeared in the medical field of the Tang Dynasty. In Chinese medicine, the crust is used to treat urinary dysfunction, and the fruits are used to treat fever. In Ayurveda, the fruits are also used to treat epilepsy, lung diseases, asthma, cough, and urinary retention. Starting from these traditional uses, the present paper evaluated the latest in vivo and in vitro pharmacological studies that demonstrated the molecular mechanisms which confirmed ethnopharmacological uses. However, a limiting aspect of this paper is the lack of clinical trials in human subjects. In the future, they are needed to complete the pharmacological properties and to pave the way for new pharmaceutical forms based on natural compounds with proven therapeutic effects. Improvements in control standards are also needed for future pharmacological studies that include *B. hispida*. In our work, they are relative, phytochemical compounds being identified only by high-performance liquid chromatography (HPLC). Another limiting aspect is represented by the antioxidant action of this plant which has been researched only in vitro, which does not guarantee the same effect on in vivo experimental models. Also, in future studies, the bioavailability, pharmacokinetics, mechanism of action, and study of the activity relationship of the identified and isolated pure phytochemicals should be analyzed, to better understand the reported biological actions.

Although experimental toxicological studies in animals have not shown any adverse effects, no human clinical trials have been performed to demonstrate pharmacological properties or to systematically assess toxicity and safety in humans. These studies are very important for the evaluation of short- and long-term toxicity as well as clinical therapeutic efficacy. However, the results of the present study support the clinical use of *B. hispida* in modern medicine and can serve as a basis for further studies based on this plant.

## Figures and Tables

**Table 1:** Chemical phytoconstituents of *Benincasa hispida* (Thunb.) Cogn.

Compounds	Plant parts	Locality/country	References
E-2-hexenal, n-hexanal and n-hexyl formate; however, 2,5-dimethylpyrazine, 2,6-dimethylpyrazine, 2,3,5-trimethylprazine, 2-methylpyrazine, 2-ethyl-5-methylpyrazine	Fruit	Taipei, Taiwan/China	[30]
Cucumisin-like protease	Sarcocarp	Kagoshima/Japan	[32]
Triterpenes, sterols, flavonoid C-glycoside, benzyl glycoside, alnusenol, multiflorenol	Fruit	Kyoto/Japan	[33]
Osmotin-like protein	Seeds	New York/USA	[34]
Chitinase	Seeds	New York/USA	[35]
Astilbin, catechin, naringenin	Fruit	Hainan/China	[31]
Di-2-ethylhexyl phthalate	Fruit	Hainan/China	[36]
W-sitosterol, V-amyrin, quercetin	Stem	Visakhapatnam/India	[37]
*β*-Carotene	Fruit	Faisalabad/Pakistan	[16]
Tryptophan	Fruit	Gwalior/India	[38]
Linoleic, palmitic, oleic, and stearic acids	Fruit	Temerloh, Pahang/Malaysia	[39]
Acetoin, octanal, nonanal	—	Mumbai/India	[40]
*α*-Tocopherol, *δ*-tocopherol, linoleic acid, *β*-sitosterol, campesterol, stigmasterol, *Δ*^5^-avenasterol	Fruit	Serdang, Selangor/Malaysia	[41]
Galactose, glucose, xylose, sorbose	Peel	Karnataka/India	[42]
Linoleic acid, linolenic acid	Seeds	Serdang, Selangor/Malaysia	[43]
Myristic acid, palmitoleic acid, oleic acid, linoleic acid, stearic acid, *α*-linolenic acid, palmitic acid, other saturated and unsaturated fatty acids	Seed oil	Serdang, Selangor/Malaysia	[44]
3*α*,29-O-di-trans-cinnamoyl-D:C-friedooleana-7,9(11)-diene, oleanolic acid 28-O-*β*-D-xylopyranosyl-[*β*-D-xylopyranosyl-(1 → 4)]-(1 → 3)-*α*-L-rhamnopyranosyl (1 → 2)-*α*-L-arabinopyranoside, oleanolic acid 28-O-*β*-D-glucopyranosyl-(1 → 3)-*β*-D-xylopyranosyl-[*β*-D-xylopyranosyl-(1 → 4)]-(1 → 3)-*α*-L-rhamnopyranosyl-(1 → 2)-*α*-L-arabinopyranoside, multiflorenol, isomultiflorenyl acetate, stigmasterol, stigmasterol 3-O-*β*-D-glucopyranoside, *α*-spinasterol, *α*-spinasterol 3-O-*β*-D-glucopyranoside, *β*-sitosterol, daucosterol, arbutin, nicotinic acid, (+)-pinonesinol, ethyl *β*-D-glucopyranoside	Fruit	Jinghong/China	[45]
Phloem lectin-like protein	Exudate	Fukuoka/Japan	[46]
Linoleic acid, palmitic acid, oleic acid, stearic acid	Seeds	Rambagh, Allahabad/India	[47]
Gallic acid	Fruit	Kota Bharu/Malaysia	[15]
Lupeol	Seeds	Mumbai/India	[27]
Gallic acid, linoleic acid	Seeds	Serdang, Selangor/Malaysia	[28]
*β*-Sitosterol	Seeds	Mumbai/India	[48]
Ascorbic acid	Fruit	Kubang Kerian, Kelantan/Malysia	[49]
*β*-Carotein, ascorbic acid	Peel	Mysore/India	[17]
Polysaccharides	Fruit	Guangzhou/China	[50]
Gallic acid, catechin, epicatechin, rutin, quercetin, quercetin-3-D-galactoside, trans-ferrulic acid, oleanolic acid, ursolic acid	Fruit	Buzau/Romania	[9]

**Table 2:** Antioxidant properties of different parts or their extracts/fractions of isolated compounds.

Extract/isolated compounds	Test system	Results	References
Crude oil from seeds	DPPH ABTS TPC	DPPH: EC_50_ = 0.1 mg/mL ABTS: EC_50_ = 0.1 mg/mL Significant antioxidant effect Standards: methyl ether, fatty acids	[56]
Seeds extract	DPPH, ABTS, total phenolic content	EC_50_ = 10 − 100 *μ*g/mL Significant antioxidant effect Standards: methyl ether, fatty acids	
Methanolic and aqueous peel extracts	DPPH	EC_50_ = 10 − 100 *μ*g/mL Concentration-dependent radical scavenging activity The methanolic extract exhibited a better antioxidant effect Standard: DPPH	[29]
Aqueous seeds extract	TPC, TFC DPPH, ABTS, H_2_O_2_, linoleic acid oxidation nitrite scavenging assay	TPC: EC_50_ = 81.3 ± 1.4 *μ*g gallic acid/g TFC: EC_50_ = 486.8 ± 4.1 *μ*g catechin/g dry mass DPPH: EC_50_ = 0.6 − 3 mg/mL Concentration-dependent antioxidant activity Standards: catechin 0.05-0.5 mg/mL, BHT, ascorbic acid 10 mg/mL	[61]
Seed oil	DPPH ABTS radical scavenging assay	DPPH: EC_50_ = 0.1 mg/mL The antioxidant activity of the seed oil was lower than the catechin and BHT at the same concentration. Standard: FAME	[44]
Hispidalin	DPPH Lipid peroxidation assay	DPPH: EC_50_ = 2 − 40 *μ*g/mL EC_50_ = 40 *μ*g/mL Significant DPPH radical scavenging and inhibition of lipid peroxidation capacity Standard: methyl ether	[59]
Methanol, ethanol, aqueous peel extracts	DPPH Reducing power assay	Significant antioxidant effect Standard: acarbose 20, 40, 60, 80, 100 *μ*g.mL^−1^	[17]
Seed extract	DPPH ABTS	Significant antioxidant effect Standard: FAME	[28]
Polysaccharides of fruit extract	DPPH	EC_50_ = 0.98 mg/mL Significant antioxidant effect Standard: glucose	[50]

Abbreviations: TPC: total phenolic contents; TFC: total flavonoid contents (TFC); ABTS: 2, 2′-azinobis (3-ethylbenzothiazoline-6-sulfonic acid); DPPH: 2,2-diphenyl-1-picrylhydrazyl free radical-scavenging ability; BHT: antioxidant butylated hydroxytoluene; FAME: fatty acid methyl ester; EC_50_: the half-maximal effective concentration.

**Table 3:** Antimicrobial, anthelmintic, and larvicidal effects of different parts or their extracts/fractions or isolated compounds.

Extract/isolated compounds	Dose/concentration model (*in vitro/in vivo*)	Results/mechanisms	References
*Antimicrobial effects*			
Methanolic whole plant extract	*Pseudomonas aeruginosa* *Vibrio parahaemolyticus* *In vitro* Standard: DMSO	IC_50_ = 500 *μ*g/disc Zone of inhibition = 6 mm	[70]
Hispidalin	*Staphylococcus aureus*, *Escherichia coli*, *Pseudomonas aeruginosa*, and *Salmonella enterica*; Fungi: *Penicillium chrysogenum*, *Fusarium solani*, *Aspergillus flavus, Colletotrichum gloeosporioides* *In vitro* Standard: acetoin (0.01–20 *μ*g/*μ*l)	Antibacterial: MIC = 30 − 120 *μ*g/mL, Antifungal: MIC = 100 − 200 *μ*g/mL	[59]
Aqueous peel extract	*Staphylococcus aureus*, *Micrococcus luteus*, *Escherichia coli*, *Klebsiella pneumoniae* *In vitro* Standard: DMSO 150 *μ*L	Antibacterial: MIC = 6.1 − 14.5 *μ*g/mL	[11]
*Anthelmintic effect*			
Ethanolic seed extract	*Pheretima posthuman/in vitro* Standard: phenytoin sodium	IC_50_ = 20, 40, and 60 mg Dose-dependent anthelmintic effect	[72]
*Larvicidal effect*			
Phloem lectin-like protein from the exudate	*Samia ricini* larvae/*in vitro* Standards: Precision Plus Protein™^,^ serum albumin	↑ inhibitory activity against the larvae Dose: 70 *μ*g/g	[46]

Abbreviations: IC_50_: value concentration that inhibits cell growth by 50%; MIC: minimum inhibitory concentration.

**Table 4:** Cytotoxic and anticancer effects of various parts of *B. hispida* extracts/fractions.

Extract/isolated compounds	Model dose/concentration	Results/mechanisms	References
Aqueous seed extract	HUVECs, NIH/3T3 cells/*in vitro* Male C57BL/6 mice/*in vivo* IC_50_ = 20 − 800 *μ*g/mL Standard: NNGH	No cytotoxicity on HUVECs, NIH/3T3 cells decrease bFGF-induced angiogenesis in mice	[84]
Fruit, seed, root proteins	HeLa, K-562 cells/*in vitro* IC_50_ of fruit, seeds root extract = 44, 40-50 *μ*g/mL IC_50_ = 10 − 1000 *μ*g/mL in *Artemia salina* IC_50_ = 10–50 *μ*g/mL on HeLa, K-562 cells Standards: lysozime, tyrosine, carbonic anhydrase, ovalbumin, albumin	Decrease cell proliferation by 28.50-36.80%	[24]
Aqueous extract	HUVECs cells/*in vitro* IC_50_ = 1–20 *μ*g/mL on high glucose (25 mM) Standards: glucose 25 mM, glucose and ABH 5 *μ*g/ml, 20 *μ*g/ml	Decrease cell adhesion molecules activation, Decrease ROS, NF-*κ*B Decrease inhibiting monocyte adhesion	[57]
Methanolic/whole plant extract	*Artemia salina/in vitro* IC_50_ = 45.186 *μ*g/mL Standard: DMSO, vincristine sulphate 0.91 *μ*g/mL	Increase cytotoxic effect concentration-dependent	[70]

Abbreviations: IC_50_: value concentration that inhibits cell growth by 50%; bFGF: basic fibroblast growth factor; ROS: reactive oxygen species; NF-*κ*B: nuclear factor kappa-light-chain-enhancer of activated B cells; NNGH: *N*-isobutyl-*N*-(4-methoxyphenylsulfonyl)-glycylhydroxamic acid.

**Table 5:** Gastrointestinal protective effects of different parts or their extracts/fractions of *B. hispida*.

Gastroprotective	Model/dose/concentration	Mechanisms	References
*Antiulcer effect*			
Fresh juice, petroleum ether, alcoholic/fruits extract	Aspirin plus restraint, serotonin-induced ulcers, indomethacin plus histamine Swimming stress Mice/*in vivo* Dose: 1 ml/mouse	↓ulcer index formed by several ulcerogenic	[13]
Fresh juice, ethanol, petroleum ether extracts (5% v/v)	Aspirin plus restraint, swimming stress, indomethacin plus histamine, and serotonin-induced ulcers Rats and mice/*in vivo* Fresh juice (1-4 mL/animal, p.o.), Dose: ethanol extract 12, 24, and 48 mg/kg, p.o. Dose: petroleum ether extract 0.75, 1.5, 3 mg/kg, p.o.	Dose-dependent anti-ulcerogenic effect The fresh juice treatment for 3 months did not change the indices (i.e., WBC, RBC counts HCT, HB, MCV, MCH urea, and sugar) No behavioural changes in experimental animals.	[13]
Petroleum ether, methanol/fruits extract	Pylorus ligated (PL) gastric ulcers, ethanol-induced gastric mucosal damage, cold restraint-stress- (CRS-) induced gastric ulcer Rats/*in vivo* Dose: 300 mg/kg, p.o.	↓ulcer index ↓MDA ↓vascular permeability ↑ CAT	[58]
Fruit extract	Indomethacin-induced gastric ulcer Rats/*in vivo* Dose: 1 mL/kg, p.o.	↓ulcer index, ↓MDA ↓SOD, ↓vitamin C	[23]
Hydromethanol, aqueous ripe fruit, ethyl acetate extracts	Ranitidine (5 mg/kg) induced hypochlorhydria Rats/*in vivo* Dose: 20 mg/kg, p.o./alternative days for 14 days	The aqueous extract showed better effects: ↑antioxidant status, ↑pepsin, ↑vitamin C, ↑chloride in gastric juice	[60]
Fruit extract with the whole plant of *Fumaria vaillantii* Loisel (1 : 1)	Ranitidine (5 mg/kg) induced hypochlorhydria Rats/*in vivo* Dose: 20 mg/kg, p.o.	↑iron levels in serum, ↑pepsin, ↑gastric juice chloride level and liver ↑blood haemoglobin level	[85]
Fruit juice	Prospective pilot study Dyspeptic patients (*n* = 20) (baseline between 30-45 days); age 18-45 years; 200 mL single-dose every morning in empty stomach for thirty days	↓pain ↓belching retrosternal burning ↓nausea ↓bowel habits	[86]
*Antidiarrheal effect*			
Methanolic fruit extract	Castor oil-induced diarrheal, PGE2-induced, enter pooling and charcoal meal models Rats/*in vivo* Dose: 200, 400, and 600 mg/kg, orally by gavage	Dose-dependent antidiarrheal effect ↓PGE2- induced, enter pooling ↓gastrointestinal motility	[90]
Methanolic fruit extract	Castor oil, charcoal meal, and antienter pooling models in rats/*in vivo* Dose: 200, 400, 600 mg/kg, p.o.	↓ activity against castor oil-induced diarrhoea; ↓PGE2 induced enter pooling ↓gastrointestinal motility	[90]

Abbreviations and symbols:↑(increased); ↓(decreased); WBC: white blood cells; RBC: red blood cells; HCT: hematocrit; HB: haemoglobin; MCV: mean corpuscular volume; MCH: mean corpuscular haemoglobin concentration; MDA: malondialdehyde; CAT: catalase; SOD: superoxide dismutase; PGE2: prostaglandin E2.

**Table 6:** Other pharmacological activities of *Benincasa hispida* (Thunb.) Cogn.

Extract/isolated compounds	Model dose/concentration	Results/potential mechanisms	References
*Lipid-lowering effect*			
Hexane, chloroform, ethyl acetate/aqueous fruit extract	3T3-L1 cells/*in vitro*	Hexane extract: ↓adipocyte differentiation, ↓PPAR*γ*, ↓C/EBP*α*, ↓leptin gene expression, ↓lipids accumulation, ↑releasing of glycerol, ↑ triglycerides	[97]
*Antidiabetic effect*			
Methanolic/stem extract	Alloxan-induced diabetes Rats/*in vivo* Dose: 50,100, 200 mg/kg p.o.	↓blood glucose level dose-dependent	[37]
Chloroform/fruits extract	Alloxan-induced diabetes Rats/*in vivo* Dose: 250, 500 mg/kg p.o.	Dose-dependently ameliorated the disorders in the metabolism of lipids in diabetic mice	[91]
Ethanol, hexane, ethyl ethanoate/leaf extract	STZ-induced diabetes Mice/*in vivo* Dose: 0.2-1 g/kg, i.p	Ethanol, ethyl ethanoate extracts: ↓blood glucose level	[22]
*Antiobesity effect*			
Methanolic fruit extract	Mice/*in vivo* Dose: 0.2-1 g/kg, i.p.	Anorexic activity ↓food intake	[96]
*Antiageing of skin*			
Petroleum ether, chloroform, ethyl acetate, methanol/dried fruit pulp extract	Stratum corneum of human skin and dansyl chloride fluorescence models *In vitro*	Cream prepared from the fruit extract showed the significant antiageing effect	[12]
*Effects on other diseases*			
Fruit methanol extract	Antigen-antibody induced reaction in rats exudate cells/*in vitro*	↓histamine release, anti-inflammatory effect Triterpenes, sterols, multiflorenol, alnusenol exerted better inhibitory effects	[33]
Methanolic fruits extract	Histamine and acetylcholine-induced bronchospasm Guinea pigs/*in vivo* Dose: 50, 200, 400 mg/kg, p.o.	Bronchodilator effect: dose-dependent protection against histamine and acetylcholine-induced bronchospasm	[115]
Petroleum ether, methanolic/fruits extract	Histamine stimulated paw oedema carrageenan- stimulated paw oedema cotton pellet stimulated granuloma Rats/*in vivo* Dose: 300 mg/kg, p.o.	↓histamine release Anti-inflammatory effect	[58]
Juice	Isolated rat aortic ring/*in vitro* Cultured porcine endothelial cells/*in vitro* Rats/*in vivo* Dose: 0.4–1.6 mL/kg, i.v.	Antihypertensive effect dose-dependent ↓blood pressure ↑relaxation, ↓contraction of isolated rat aortic ring ↑NO in cultured porcine aortic endothelial cells	[116]
Methanolic fruit extract	Renal ischemia/reperfusion injury model Rats/*in vivo* Dose: 500 mg/kg/day, p.o. for 5 days	Nephroprotective ↓MDA, ↑SOD, CAT, ↑GSH	[117]
Ethanolic seeds extract	Ethylene glycol induced chronic Hyperoxaluria model Rats/*in vivo* Dose:250, 500 mg/kg, p.o. for 35 days	Nephroprotective ↓ urinary oxalate, ↓endogenous oxalate synthesis; ↓urinary protein excretion, ↓kidney oxalate and calcium; ↓elevated serum levels of sodium, creatinine, calcium, phosphorus	[118]
*Neuroprotective effects*			
Fruit juice	Morphine addiction model Mice/*in vivo* Dose: 1 mL/mouse, p.o.	The development of morphine addiction prevented along with the suppression of opioid withdrawal symptoms	[113]
Methanolic fruit extract	Spontaneous motor, muscle relaxant, antihistaminic effect and barbiturate induced hypnosis models Mice, rats, and guinea pigs/*in vivo* Dose: 200-3000 mg/kg, p.o.	↑ barbiturate induced hypnosis ↑ antihistaminic activity	[114]
Fruit methanol extract	Pentylenetetrazole, strychnine, picrotoxin, and maximal electro seizures model Rats/*in vivo* Dose: 0.2-1 g/kg, p.o.	Dose-dependent anticonvulsant activity	[98]
Methanolic fruit extract	Acetic acid-induced writhing and hot plate Model Mice/*in vivo* Dose: 200, 400, 600 mg/kg, p.o.	Dose-dependent analgesic effect	[111]
Aqueous pulp extract	Colchicine-induced Alzheimer's model Rats/*in vivo* Dose: 100-450 mg/kg, p.o.	↑SOD, ↑CAT, ↑GSH, ↓LPO dose-dependent	[16]
Ethanolic seed extract	Rats/*in vivo* Dose: 250, 500 mg/kg, p.o.	Dose-dependent analgesic and antipyretic effects	[26]
Methanolic fruit extract	Marble-burying and motor coordination tests Mice/*in vivo* Dose: 200, 400, 600 mg/kg, p.o.	Significant dose-dependent anticompulsive effect	[38]
Methanolic leaf extract	Acetic acid-induced writhing Mice/*in vivo* Dose: 50, 100, 200, 400 mg/kg, p.o.	Dose-dependent analgesic effect	[112]
Fruit peel methanolic extract	Egg albumin-induced inflammation in rats; acetic acid-induced writhing, formalin-induced pain, hot plate-induced, and pentylenetetrazol-induced convulsions Mice/*in vivo* Dose: 50, 100, 200, 400 mg/kg, p.o.	Dose-dependently (0.25-1.5 g/kg) inhibited acetic acid-induced writhing, formalin-induced pain licking, and hot plate-induced pain in mice. Significantly inhibition of egg albumin-induced inflammation in rats and pentylenetetrazol-induced convulsion in mice	[62]
Ethanolic seed extract	Anticonvulsant activity Mice/*in vivo* Dose: 250, 500 mg/kg, p.o.	Dose-dependent anticonvulsant effects	[72]
Methanolic fruit extract	TST and FST model Mice/*in vivo* Dose: 50, 100, 200 mg/kg, p.o.	Dose-dependent antidepressant effect possibly through GABAergic involvement.	[110]
Petroleum ether, methanolic, aqueous/fruit extracts	Motor coordination, locomotor, cognitive behaviour, anxiolytic, haloperidol-induced catalepsy, and anticonvulsant models Mice/*in vivo* Dose: 100, 200, 400 mg/kg, p.o.	Dose-dependent anxiolytic, analgesic, and nootropic activity	[107]

## Data Availability

The data supporting this review are from previously reported studies and datasets, which have been cited. The processed data are available from the corresponding author upon request.

## Abbreviations

ABTS: 2,2′-azino-bis(3-ethylbenzothiazoline-6-sulfonic acid
ALP: Alkaline phosphatase
bFGF: Basic fibroblast growth factor
CAT: Catalase
C/EBP*α*: CCAAT enhancer-binding protein alpha
DPPH: 2,2-diphenyl-1-picrylhydrazyl
HPLC: High-performance liquid chromatography
HUVECs: Human umbilical vein endothelial cells
GSH: Reduced glutathione
ICAM-1: Intercellular adhesion molecule
IL-6: Interleukin 6
LC_50_: Median lethal concentration
LPO: Lipid peroxidation
MCV: Mean corpuscular volume
MDA: Malondialdehyde
NIH/3 T3: Normal fibroblast cells
NO: Nitric oxide
PGE2: Prostaglandin E2
PPAR*γ*: Peroxisome proliferator-activated receptor-gamma
ROS: Reactive oxygen species
SGOT: Serum glutamic oxaloacetic transaminase
SGPT: Serum glutamic pyruvic transaminase
SOD: Superoxidase dismutase
STZ: Streptozotocin
TPC: Total phenolic content
TNF-*α*: Tumour necrosis factor-alpha.
